# Warthin-Starry Staining for the Detection of *Helicobacter pylori* in Gastric Biopsies

**DOI:** 10.21315/mjms2018.25.4.9

**Published:** 2018-08-30

**Authors:** Wirda Indah Farouk, Nur Hidayah Hassan, Teh Rasyidah Ismail, Intan Sufinaz Daud, Fazarina Mohammed

**Affiliations:** 1 Universiti Kuala Lumpur, Institute of Medical Science & Technology (UNIKL MESTECH), 43000 Kajang, Selangor, Malaysia; 2 Pathology Department, Universiti Kebangsaan Malaysia Medical Center (UKMMC), 56000 Cheras, Wilayah Persekutuan Kuala Lumpur, Malaysia

**Keywords:** H. pylori, Warthin-Starry, sensitivity, specificity, positive predictive value, negative predictive value

## Abstract

**Background:**

Warthin-Starry (WS) staining is an ancillary stain used in the detection of *Helicobacter sp.*, spirochaete and other microorganisms in tissue sections. The present study aimed to determine the validity of WS stain in the confirmation of *H. pylori* diagnosis in gastric biopsies in comparison with anti*-H. pylori* immunohistochemistry (IHC) staining.

**Methods:**

This study involved 104 cases of gastric biopsies that were previously subjected to WS staining. All cases involved retrieval of formalin-fixed paraffin-embedded (FFPE) gastric biopsies that were re-cut, subjected to anti*-H. pylori* IHC staining and reviewed blindly by a pathologist. The sensitivity, specificity, positive predictive value (PPV) and negative predictive value (NPV) of WS as compared to IHC were calculated.

**Results:**

In this study, WS stain was less sensitive in detecting *H. pylori*. The sensitivity, specificity, PPV and NPV for WS stain were 50.0%, 92.4%, 79.2% and 76.3%, respectively.

**Conclusions:**

The sensitivity of WS stain in the histopathology laboratory was lower than that described previously. Several external factors that might influence the results were identified. However, sufficient information on patients’ history of treatment and medication would be required for the diagnosis or confirmation of the presence of *H. pylori* in gastric biopsies by WS staining.

## Introduction

*Helicobacter pylori* is a microorganism that can be found in the stomach and is known to be pathogenic. The bacteria are Gram-negative, motile and microaerophilic such that they can withstand the acidity of the stomach for survival. The species inhabit the gastric mucosa, which consists of a mucus layer that provides protection for *H. pylori* from the harsh environment of the stomach. The infected areas present microscopic features of gastritis that showed an increased number of chronic inflammatory cells such as lymphocytes and neutrophils in the lamina propria of the stomach. Prolonged or untreated infection may predispose to gastric carcinoma and mucosa-associated lymphoid tissue (MALT) lymphoma (1, 2). *H. pylori* is known to have an oncogenic role, which is exhibited during the inflammatory process that involves specific genes and increased the virulence of *H. pylori* to cancer (3).

Generally, the prevalence of *H. pylori* infection varies demographically. Although the infection may affect all ages, adults ≥ 60 years of age were rather prone to the infection (4). *H. pylori* can cause infection by multiple routes; however, the ingestion of bacteria is speculated as primary mode of transmission from one person to another (5), which might be attributed to the consumption of bacteria-tainted water and failure to practice proper hygiene (6). The prevalence of the bacteria is higher in developing countries as compared to developed countries due to low economic and academic backgrounds. These populations practice poor self-hygiene and lack awareness about the infection and the potential mortality (5).

Currently, various diagnostic approaches are available for the detection of *H. pylori* in the gastric region. The tests involve non-invasive and invasive methods. The invasive methods include urease breath test (UBT) and saliva and stool antigenic test, while the non-invasive tests are convenient to the affected person and cost-effective with high sensitivity and specificity. UBT has 81%–100% sensitivity and 80%–98% specificity, whereas stool antigenic assay has 91%–93% sensitivity and specificity (7) with less diagnostic turnaround time.

Invasive tests can cause irritation and be traumatic to the patient. Nevertheless, they provide high sensitivity and specificity. Moreover, these tests include histopathology examination (HPE), culture and sensitivity testing as well as rapid urease test (RUT) that require sampling of the gastric tissue by endoscopy. It is one of the invasive methods that require expert assessment of gastric mucosa of the infected site. Thus, the clinician conducting endoscopy needs to determine the correct site of infection by the physical appearance of gastric mucosa, wherein the affected areas could be red, nodular and oedematous. Nowadays, several indicators can be used for the true determination of the site of *H. pylori* inhabitation; for instance, phenol red chromoendoscopy has a high diagnostic accuracy and provides an accurate determination of bacterial colonisation (8). These tests can produce reliable results by direct identification of the bacteria in the gastric tissue.

In HPE testing, *H. pylori* can be detected in routine haematoxylin and eosin (H&E) staining. However, the detection should also be supported by special staining, as the bacteria might be missed during interpretation, primarily, the fragmented as well as coccoid *H. pylori* bacteria due to treatment intervention. The ancillary stains that can be used in the detection of *H. pylori* are Giemsa, Warthin-Starry (WS), Genta and modified methylene blue. Currently, the optimal stain for the detection of *H. pylori* is unknown, and hence, might depend on the availability in terms of expertise, cost and time in each laboratory.

In a usual histopathology laboratory setting, routine H&E can detect *H. pylori* bacteria in the tissue with severe infection. Nevertheless, for low-density bacterial colonisation with features of chronic gastritis might require specific staining for the identification of *H. pylori* in gastric tissue samples. In this study, WS was used for the detection of *H. pylori* in paraffin-embedded tissue sections, as well as, other microorganisms, such as fungus and spirochaetes.

The present study aimed to determine the validity of WS stain in detecting *H. pylori* in gastric biopsies using immunohistochemistry (IHC) staining as a gold standard.

## Methods

This is a descriptive, cross-sectional study using archival histopathological material from paraffin-embedded gastric biopsies samples. A total of 104 cases of formalin-fixed paraffin-embedded (FFPE) gastric biopsies, previously sectioned and stained with WS, were retrieved from the laboratory archive. The gastric resection specimens and biopsies that were subjected to Giemsa staining for the detection of *H. pylori* were excluded from this study. The demographic information was also collected from the medical record to determine the associations with the *H. pylori* infection.

### Warthin-Starry Staining Method

The previous WS staining was performed using Artisan DakoCyto machine (ADC) with ready-to-use Warthin-Starry (WS) staining kit (Code No: AR181, Dako, Denmark), pre-filled in staining cartridges. The slides were deparaffinised manually by two rinses in xylene, followed by dehydration in downgraded series of alcohol (100%, 90%, 80% and 70%), and then, running tap water. The slides were loaded in ADC for automated staining steps. A volume of 1 mL of pre-treatment solution from the kit was dispensed and incubated for 110 s at 60 °C. Subsequently, 1 mL of silver nitrate was added and incubated for 200 s at 60 °C. Then, 1 mL hydroquinone was dispensed and the mixture incubated for 105 s at 60 °C. Finally, the slides were washed six times with Artisan™ 50× Wash Solution (Code No: AR102, Dako) that was diluted to a 1× working solution with deionised water. Before coverslipping with Coverseal™ -D mounting medium (Code No: FXD117, Cancer Diagnostics Inc., USA), the slides were dehydrated with a graded series of alcohol (70%, 90%, 100% and 100%) and cleared with two changes of xylene. Positive and negative WS staining were differentiated by the presence (positive) and absence (negative) of black silver stain of the bacteria on the gastric mucosal surface against brown and yellow to dark yellow on the tissue background.

### IHC Staining Method

The gastric biopsy samples, in the form of paraffin-embedded tissue, were sectioned into 3-μm-thick sections and mounted on silanised slides (Platinum Pro White, Product No: PRO-01, Matsunami, Japan) prior staining with IHC method to determine the validity of previous WS staining results. The manual IHC staining was performed using the protocol from EnVision™ FLEX Mini Kit, High pH (Code No: K8023, Dako). The washing steps between each reagent were performed using EnVision™ FLEX Wash Buffer 20× (Code No: 8007, Dako) or Tris-buffered saline (TBS) three times each for 3 min. The 1× DAB-containing substrate working solution was prepared by diluting the 50× concentrated EnVision™ FLEX DAB+Chromogen with EnVision™ FLEX Substrate Buffer (Code No: K8023, Dako). The staining continued with slides pre-heated on a 60 °C hot plate. Deparaffinisation and pre-treatment steps were performed in Decloaking Chamber™ NexGen (Ref No: DC2012- 220V, Biocare Medical, California) using the EnVision™ FLEX Target Retrieval Solution, High pH (Code No: K8004, Dako Denmark) at 110 °C for 30 min, followed by cooling at room temperature (RT) for 20 min. Then, the slides were rinsed under running tap water for 3 min and briefly washed with TBS, followed by incubation in EnVision™ FLEX Peroxidase-Blocking Reagent (Code No: DM821, Dako) for 5 min. Subsequently, the slides were incubated with ready-to-use polyclonal rabbit anti-*H. pylori* primary antibody (Lot No: 20026310) for 30 min at RT and washed and the corresponding EnVision™ FLEX HRP (Code N: K8023, Dako Denmark) for 20 min at RT. The immunogenic complex was labelled with 1× DAB solution for 5 min and counterstained with haematoxylin 2 (REF 7231, Thermo Scientific, USA) for 15 s. Then, the slides were washed under running tap water, dehydrated and cleared with 80%, 90%, 100% and 100% alcohol solutions and two changes of xylene. Finally, the slides were cover-slipped with DPX mounting medium (Cat. No.: 100579, Merck Millipore, Germany) and examined qualitatively by a pathologist blinded to the samples. Positive *H. pylori* showed brownish discolouration of bacteria by chromogen, while the negative *H. pylori* did not show any brownish discolouration with purple to blue background staining by haematoxylin.

#### Calculation of sensitivity, specificity, positive predictive value and negative predictive value

Sensitivity, specificity, positive predictive value (PPV) and negative predictive value (NPV) were calculated based on the frequency of WS and IHC staining. The parameters were calculated using the following formulae:

**Table:** 

Sensitivity	: [TP / (TP+FN)] * 100	Note: TP = True positive TN = True negative FP = False positive FN = False negative
Specificity	: [TN / (TN+FP)] * 100	
PPV	: [TP / (TP+FP)] * 100	
NPV	: [TN / (TN+FN)] * 100	

#### Statistical analysis

All data recorded were analysed statistically using the Statistical Package for the Social Sciences (SPSS) software version 23.0. The significant difference between the expected and observed frequencies was computed by Pearson’s chi-square (for independence) analysis. *P*-value < 0.05 was considered statistically significant.

## Results

### Demographic Analysis of the 104 WSStained FFPE Gastric Biopsy Samples

The demographic profile of the retrieved sample was collected from the medical record and displayed in Table 1. Among the 104 WS-stained gastric biopsies registered, the patients’ age ranged from 10–85 (mean, 56.24) years. The cohort consisted of 63.5% males and 36.5% females. About 47.1% of the samples were from Malay, 37.5% were Chinese and 15.4% were other ethnicities.

### IHC of the 104 Previously WS-Stained Cases

Among the 104 previously WS-stained cases, 24 (23%) were positive, and the remaining 80 (77%) slides were negative (Table 2). In order to determine the validity of the WS staining results, the FFPE blocks from WS-stained cases were further re-sectioned and subjected to IHC staining. The 19/24 WS-positive slides, were positive for IHC stain, and 19/80 WS-negative slides were found to be IHC positive (19 true positives and 19 false negative results). As a result, 38 positive cases of *H. pylori*-infected gastric biopsies were confirmed with IHC stain.

### Demographic Association with H. pylori Infection

Table 3 showed the demographic association with *H. pylori* infection among the tested slides. Pearson’s chi-square analysis found that Chinese race had the highest (15) significant number of positive cases of *H. pylori* as compared to other races (*P* = 0.001). Other variables were not significantly associated with the infection.

**Figure 1:**
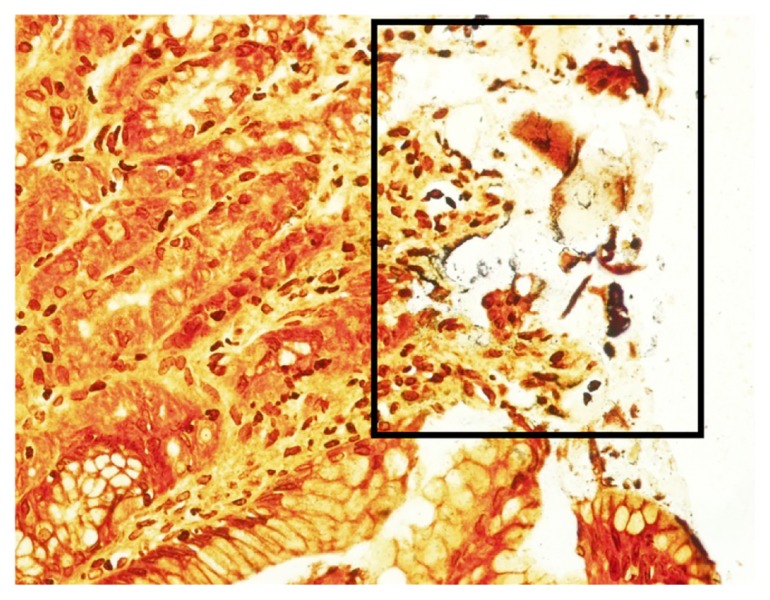
Area of granular staining at the mucosal surface area (box) of negative WS staining that might be misinterpreted as a positive *H. pylori* infection. The lamina propria showed minimal inflammatory infiltration that might suggest an association with *H. pylori* infection (40×)

**Figure 2:**
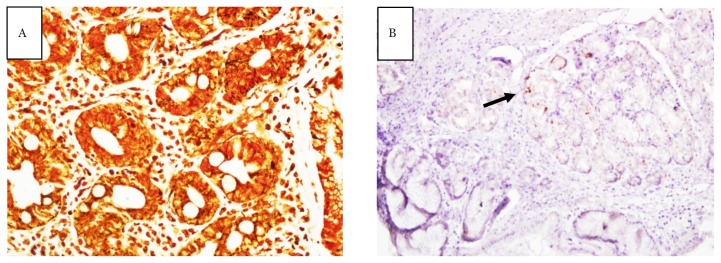
Slide stained with WS staining at 40× magnification (A) and slide stained with IHC at 10× magnification (B); both were from the same gastric biopsy tissue. Bacteria appeared as fragmented and misreported as negative WS staining. Confirmation with the gold standard staining (IHC) showed positive *H. pylori* infection (arrow)

The slides were carefully reviewed to determine the nature of discrepancies. The observation resulted in five positive WS staining with negative IHC staining. These cases were considered to be false positive WS staining, probably due to the granular background WS staining and misinterpreted as bacteria (Figure 1). An additional number of 19 cases with negative WS staining was found to be positive IHC stain. These cases were considered to be false negative WS staining probably due to the fragmented and scarce distribution of bacteria in gastric mucosal tissue (Figure 2).

#### Sensitivity, specificity, positive predictive value and negative predictive value analysis

Sensitivity, specificity, PPV and NPV were calculated based on the formula explained in the methodology section. The sensitivity, specificity, PPV and NPV for WS staining were 50%, 92.4%, 79.2% and 76.3%, respectively (Table 4).

## Discussion

In severe infection, the *H. pylori* bacteria might be easily appreciated in the routine H&E staining. However, samples that were only suggestive of *H. pylori* infection would need a confirmatory test (WS-specific staining) to reveal the organism that might not be observed in the H&E stain. Hartman and Owens (9) reported that any gastric biopsies with at least a moderate activity and no appreciation of the bacteria would need further special staining, such as WS, to avoid overlooking the scattered organism.

In this study, the validity of WS staining was determined using IHC method as a gold standard. The IHC staining can be defined as a sensitive method applying antigen-antibody reaction principle. Tajalli et al. (10) studied the comparison between IHC and toluidine blue in detecting low-density coccoid form of *H. pylori*. On the other hand, Rotimi (11) revealed a 98% sensitivity of IHC staining in his study. Furthermore, Ryan and Lourie (12) indicated IHC as a gold standard with 100% sensitivity.

This study used a polyclonal rabbit antibody of *H. pylori* and HRP detection kit. Polyclonal antibody showed a high affinity towards the antigenic properties of the bacteria that can recognise multiple epitopes. Therefore, unlike WS that can stain other microorganisms in addition to *H. pylori*, IHC solely stains the bacteria with properties of an ultimate gold standard method for validity testing. However, other studies revealed that WS was a sensitive stain that confirmed the presence of *H. pylori*. This phenomenon was in agreement with the study by Pandya et al. (13). The WS values of sensitivity, specificity, PPV and NPV were scored as 100%, 84.5%, 59.1% and 100%, respectively. This also supported by a previous study by Patnayak et al. (14), which scored 100% of WS sensitivity. Most of these studies used manual WS staining procedure, wherein the silver coating made the bacteria large and identifiable. Therefore, subtle cases of low density or fragmented *H. pylori* could be noted easily that increased the value of sensitivity.

Several technical factors have been discovered that contribute towards the false result of WS staining, thereby affecting the sensitivity and specificity value in this study. The cases with negative WS staining, which were found to be positive for *H. pylori* infection, were those with fragmented and weak IHC staining. The bacteria were scantily and sparsely distributed that might be overlooked during WS interpretation. The low density of bacteria might be affected by possible sampling error during endoscopy that might not represent the site of infection; the nearby site could be distant from the brim of infection and *H. pylori* colonisation.

In the untreated samples of gastric biopsies, *H. pylori* bacteria would appear spiral and distinguishable in WS staining. However, the spiral-shape may alter to coccoid shape due to antibiotic or proton pump inhibitor (PPI) intervention in post-treatment gastric biopsies. Medications such as NSAIDs and PPI are known to change the morphology and urease production of the bacteria. The study by Jekti et al. (15) revealed that the development of *H. pylori* into coccoid form initiated after 13.5 days post-antibiotic treatment. In WS staining of post-treatment samples, *H. pylori* also appeared granular in morphology, and observers might have difficulty in recognising the bacteria that are indistinguishable from inevitable background staining in WS-stained slides and may be reported as negative. Conversely, background granular WS staining might be misinterpreted as bacteria and result in false positives. Hence, skilled and experienced observers are critical for the interpretation of WS results. In addition, false-positive WS staining may be caused by non-*H. pylori* bacteria, such as other microorganisms and *spirochaetes*. These non-*H. pylori* bacteria might be acquired from the contaminated surface of slides/paraffin blocks during preparation and staining procedure or the existing pathogen on the tissue samples that were obtained from different species.

Another possible cause might occur during reproducing the tissue sectioning for further investigation that would acquire the area with bacterial colonisation. These sections indicate the initial lack of *H. pylori*; however, the acquisition of the bacterial colonies in the subsequent sections subjected to IHC could result in false negative staining. Conversely, deeper sectioning might also cause false negative result due to loss of bacteria in subsequent sections for IHC staining.

Also, there were other discrepancies observed during the evaluation of IHC results. In this study, the manual staining technique of IHC may be technically inconsistent in terms of background staining build-ups that could interfere in the detection of *H. pylori*. This phenomenon might arise due to the imperfect washing steps and second by the usage of a polyclonal antibody, which increased the chances of non-specific staining obscuring the identification of the bacteria. Moreover, the presence of artefact and debris could also contribute to the problem, and can be rectified using IHC automated staining system for the uniform outcome.

## Conclusion

In this study, the sensitivity of WS staining as lower than the previous studies due to the technical limitations. The protocol may be improved in the future by utilising an automated staining machine and a ready-to-use staining kit. Thus, the variability in staining process would be reduced by factors of human techniques. Moreover, positive control of *H. pylori*-infected tissues was appended on each of the run slides for quality assurance. In addition, external factors also contributed to low sensitivity. Moreover, the specificity of this WS staining was 92.4%.

Patient history of treatment and administered medication must be diligently retrieved for the determination of *H. pylori* association if not provided by the clinician, who requested gastric biopsies for HPE. The observers should be aware that the pre- or post-treatment samples play a major role in the accurate result of WS staining due to its ability for altering the morphology and/or being eradicated by the drugs. If treatment was inevitable, the medication might be suspended prior to sample collections.

Also, the observers’ experiences were critical for correct diagnosis. However, pathologists are still able to identify these distinctive bacteria. Good staining quality is paramount in most subtle cases. Additionally, clinicians who carry out endoscopy must also determine the infection area, of which, *H. pylori* is commonly present densely on the stomach mucosa. If the clinicians fail to identify the site (dense colonisation of *H. pylori*), the samples might represent low density or lack of bacteria, thereby affecting the laboratory results.

## Figures and Tables

**Table 1:** Distribution of patients’ information by demographic profile (*n*number of = 104)

Demographic profile		*n*	%
Gender	Male	66	63.5
	Female	38	36.5
Race	Malay	49	47.1
	Chinese	39	37.5
	Others	16	15.4
Age (years)	0–50	32	30.8
	51–100	72	69.2

**Table 2:** Frequency (*n*) and percentage of WS and IHC staining results

Routine *H. pylori* detection		IHC confirmation (gold standard)	
	
Warthin-Starry staining result		*H. pylori* infection	

		Present	Absent
Positive	24 (23%)	19	5
Negative	80 (77%)	19	61

**Total**	**104**	**38**	**66**

**Table 3:** Demographic association with *H.pylori* infection

Aspects	*H. pylori*-infected		Pearson chi-square	df	*P*

	Yes	No			
Gender					
Male	20	46	3.029	1	0.060
Female	18	20			
Race					
Malay	11	38	14.460	2	0.001
Chinese	15	24			
Others	12	4			
Age group (years)					
0–51	13	19	0.330	1	0.358
51–100	25	47			

	38 (36.5%)	66 (63.5%)			

**Table 4:** Sensitivity, specificity, positive and negative predictive value of WS staining for the detection of *H. pylori* as compared to the IHC staining

Staining method	Sensitivity (%)	Specificity (%)	PPV* (%)	NPV* (%)
Warthin-Starry	50	92.4	79.2	76.3

* PPV: Positive Predictive Value, NPV: Negative Predictive Value
